# Aceclofenac/Citronellol Oil Nanoemulsion Repurposing Study: Formulation, In Vitro Characterization, and In Silico Evaluation of Their Antiproliferative and Pro-Apoptotic Activity against Melanoma Cell Line

**DOI:** 10.3390/biomedicines11092531

**Published:** 2023-09-14

**Authors:** Mona K. Younis, Islam A. Khalil, Nancy S. Younis, Rasha R. Fakhr Eldeen, Rana M. Abdelnaby, Reem A. Aldeeb, Amal A. Taha, Doaa H. Hassan

**Affiliations:** 1 Department of Pharmaceutics, College of Pharmaceutical Science and Drug Manufacturing, Misr University for Science and Technology, 6th of October City 12566, Egypt; islamkhll@gmail.com (I.A.K.); reem.eldeeb@must.edu.eg (R.A.A.); amal.anwar@must.edu.eg (A.A.T.); doaa.hassan@must.edu.eg (D.H.H.); 2 Department of Pharmaceutical Sciences, College of Clinical Pharmacy, King Faisal University, Al-Ahsa 31982, Saudi Arabia; nyounis@kfu.edu.sa; 3 Zagazig University Hospitals, Zagazig 44519, Egypt; 4 Department of Biochemistry, College of Pharmaceutical Science and Drug Manufacturing, Misr University for Science and Technology, 6th of October City 12566, Egypt; rasha.rashid@must.edu.eg; 5 Department Pharmaceutical Chemistry, Faculty of Pharmacy, Heliopolis University, Cairo 11785, Egypt

**Keywords:** aceclofenac, citronellol, nanoemulsion, melanoma, antiproliferative, apoptosis, in silico study, cytotoxicity study, repurposing

## Abstract

Aceclofenac (ACF) is a widely used non-steroidal anti-inflammatory drug (NSAID) known for its effectiveness in treating pain and inflammation. Recent studies have demonstrated that ACF possesses antiproliferative properties, inhibiting the growth of cancer cells in various cancer cell lines. Citronellol, a monoterpenoid alcohol found in essential oils, exhibits antioxidant properties and activities such as inhibiting cell growth and acetylcholinesterase inhibition. In this study, the objective was to formulate and evaluate an aceclofenac/citronellol oil nanoemulsion for its antiproliferative effects on melanoma. The optimal concentrations of citronellol oil, Tween 80, and Transcutol HP were determined using a pseudoternary phase diagram. The formulated nanoemulsions were characterized for droplet size, zeta potential, thermophysical stability, and in vitro release. The selected formula (F1) consisted of citronellol oil (1 gm%), Tween 80 (4 gm%), and Transcutol HP (1 gm%). F1 exhibited a spherical appearance with high drug content, small droplet size, and acceptable negative zeta potential. The amorphous state of the drug in the nanoemulsion was confirmed by Differential Scanning Calorimetry, while FTIR analysis indicated its homogenous solubility. The nanoemulsion showed significant antiproliferative activity, with a lower IC50 value compared to aceclofenac or citronellol alone. Flow cytometric analysis revealed cell cycle arrest and increased apoptosis induced by the nanoemulsion. In silico studies provided insights into the molecular mechanism underlying the observed antitumor activity. In conclusion, the developed aceclofenac/citronellol oil nanoemulsion exhibited potent cytotoxicity and pro-apoptotic effects, suggesting its potential as a repurposed antiproliferative agent for melanoma treatment. In a future plan, further animal model research for validation is suggested.

## 1. Introduction

Cancer is one of the most life-threatening diseases; it causes death for millions of patients annually [1]. Cancer is defined as abnormal changes in the growth and division of normal cells, transforming them into cancer cells [2]. These abnormalities arise when genetic changes occur in specific types of genes: tumor suppressor genes, DNA repair genes, and proto-oncogenes [3]. The causes of the genetic disorders can either be inherited or initiated by certain carcinogens such as the ultraviolet rays from the sun and chemicals in tobacco smoke, etc. [4]. There are more than 100 types of cancer. Cancers are usually named for the organs or tissues where they develop [1].

Malignant melanoma is a serious type of skin cancer that arises from the malignant transformation of melanocytes. It is regarded as one of the deadliest forms of skin cancer [5]. It has a high ability to spread to other parts of the body, causing metastasis [6]. The development of melanoma is associated with both environmental and genetic factors [7]. A key environmental factor in melanoma development is strongly associated with prolonged exposure to ultraviolet (UV) radiation from the sun or other sources, such as mercury vapor lamps, germicidal lamps, tanning booths, and fluorescent and incandescent sources. These UV radiations cause DNA damage and genetic mutations [8]. Such mutations in the genes BRAF (located on chromosome 7), NRAS (located on chromosome 1), and CDKN2A (located on chromosome 9) lead to the initiation and progression of melanoma [8,9]. The incidence of melanoma has been rapidly increasing worldwide, highlighting the need for more effective and safe treatment [10]. The development of such treatment remains a major challenge. While surgery, radiation therapy, and chemotherapy are commonly used to treat melanoma [11], they are associated with significant adverse effects and limited efficacy in advanced cases [5,12]. Therefore, there is a growing need for novel therapeutic approaches that can improve patient outcomes.

One such approach is repurposing/repositioning existing drugs for alternative indications. This strategy involves identifying new uses for existing drugs that have been approved for other indications [13,14]. Drug repurposing offers several advantages over de novo drug discovery, such as reduced costs, shorter development timelines, and a higher likelihood of success in clinical trials, as the safety and pharmacokinetic profiles of the repurposed drug are already established [15]. In this context, anti-inflammatory drug repurposing is gaining attention as a new promising cancer treatment [16].

Anti-inflammatory drugs are known to modulate various signaling pathways that are involved in inflammation, cell growth, survival, and apoptosis [17]. Several epidemiological studies have denoted a strong correlation between inflammation and cancer [18]. Furthermore, studies on different NSAIDs showed a well-established antiproliferative effect on cancer cells [17]. Aceclofenac (ACF) is a non-steroidal anti-inflammatory drug (NSAID) that has been widely used for treating pain and inflammation in various conditions [19]. ACF has also been reported to have antiproliferative (the ability of a substance to inhibit the growth and multiplication of cancer cells) effects on different cancer cell lines, such as breast, colon, lung, and prostate cancer [20]. However, its use in cancer treatment remains constrained by challenges such as poor solubility (BSC II class) and limited bioavailability [21], hindering its therapeutic efficacy and necessitating innovative formulation approaches [22].

Another major concern in cancer therapy is to deliver drugs efficiently and selectively to the tumor site, while minimizing the side effects and toxicity to normal tissues [23]. To address these limitations, nano-drug delivery systems offer a promising solution for these hurdles by using nanosized materials that can enhance the solubility, stability, bioavailability, and targeting of drugs [24,25]. Among the numerous types of nano-drug delivery systems are nanoemulsions (NEs) [26], which are colloidal dispersions of nanosized droplets of one liquid dispersed in another liquid [27]. A NE system consists of oil, water, and a surfactant/co-surfactant. The role of SAAs is to stabilize the small particles through decreasing interfacial tension, preventing droplet aggregations and increasing surface area. NEs are generally classified as either oil in water (O/W) and water in oil (W/O) [28]. Nanoemulsions (NEs) have the ability to encapsulate hydrophobic drugs, enabling the development of aqueous formulations that facilitate targeted delivery to specific cells [26,29]. Nanoemulsions (NEs) offer a selective targeting towards cancer cells, enhancing the efficacy of cancer treatment while minimizing toxicity to healthy cells [24,29]. NEs can also improve the penetration and retention of drugs in the skin, which is especially beneficial for treating skin cancers [24]. In the current study, an ACF/citronellol oil NE (essential oil-based NE) was prepared. The criteria of selecting citronellol to formulate the NE were based on the anticancer effect of essential oils [30].

Citronellol is an essential oil that is found in certain plants, such as lemongrass, citronella, and palmarosa [31]. Citronellol has shown potential health benefits, such as antioxidant, anti-inflammatory, antimicrobial, and antifungal effects [32,33]. Moreover, citronellol may have anticancer effects by inducing apoptosis (cell death) in tumor cells, inhibiting angiogenesis (blood vessel formation), and modulating the immune system [31]. Citronellol has been demonstrated to have antiproliferative effects on various cancer cell lines, such as leukemia, cervical, ovarian, and breast cancer [34,35,36,37]. However, the antiproliferative activity of citronellol on melanoma cells has not been explored yet.

Therefore, this study repurposes the anti-inflammatory drug ACF as an antiproliferative agent for treating melanoma by preparing a NE containing aceclofenac/citronellol using the sonication method. The NE was evaluated to assess the size, charge, physical stability, and in vitro drug release profiles. Furthermore, in silico docking studies and target prediction analyses were performed to evaluate the antitumor activity and the possible synergistic antiproliferative effects on melanoma cells resulting from the presence of both aceclofenac and citronellol in the formulated NE.

## 2. Materials and Methods

### 2.1. Materials

Aceclofenac (ACF) was kindly provided by Bristol Mayers Squibb (Cairo, Egypt). Citronellol oil, Tween 80, and highly purified 98 diethylene glycol monoethyl ether (Transcutol^®^HP) were purchased from Alpha Chemika Company (Mumbai, India). Potassium dihydrogen phosphate and disodium hydrogen phosphate were supplied from El Nasr Pharmaceutical Chemicals (Cairo, Egypt). All solvents were analytical grade and supplied by Sigma Aldrich (Taufkirchen, Germany). The Human Melanoma cell line (A375) was obtained for cell culture from Nawah Scientific Inc., (Cairo, Egypt). DMEM media, streptomycin, penicillin, and heat-inactivated Fetal Bovine Serum (FBS) were purchased from Thermo Fisher Scientific (Pittsburgh, PA, USA).

### 2.2. Solubility Studies

The solubility of ACF in different media (Citronellol, Tween 80, Transcutol HP, and water) was studied. A large excess of ACF (3.5 g) was taken in centrifuge tubes and a 5.0 g media phase was added. The mixture was shaken using a vortex mixer (Heidolph Instrument, D-91126 Schwabach, type RZR 2021, Bavaria, Germany) for 15 min, followed by storage at room temperature. After 24 h, the resulting solution was subjected to centrifugation at 3000 rpm for 5 min to separate the clear supernatant. A suitable sample solution was collected and diluted with absolute methanol. The diluted sample was filtered using Whatman 102 filter paper and further diluted with absolute methanol for the determination of the drug concentration in the saturated solution. Analysis of the samples was performed using a UV spectrophotometer (DU 640; Beckman, CA, USA) at a wavelength of 276 nm, with methanol as the blank reference [38].

### 2.3. Estimation of Pseudoternary Phase Diagram

A mixture of Tween 80 and Transcutol HP was prepared in a 1:1 to 1:9 ratio and combined with the citronellol oil in a different volume ratio (from 1:9 to 9:1). Then, mixtures were screened for miscibility by a dropwise addition of water until reaching 50 mL [39]. The results were plotted on the Golden Software Grapher program (Version 8.1.388) to detect the emulsification regions.

### 2.4. Preparation of Aceclofenac/Citronellol Oil NE (Sonication Technique)

Based on the solubility study and pseudoternary phase diagram in the previous steps, 30 mg of ACF was found to be sufficiently soluble in the selected system mixtures of oil, surfactant, and co-surfactant. After mixing the drug, water was added drop by drop with stirring until the complete volume was 10 mL. Then, sonication was applied for 5 min with a sonicator (Ultrasonic LC 60 H, Elma, Germany). All formulations were stored at room temperature [40].

### 2.5. Characterization of Aceclofenac/Citronellol Oil NE

#### 2.5.1. Droplet Size and Surface Charge Analysis

The droplet size distribution and zeta potential of the prepared formulations were analyzed using the Malvern Zetasizer 2000 with backscatter detection conditions of 173°; temperature 25 °C; and refractive index 1.330 [41].

#### 2.5.2. Thermodynamic Stability Tests

##### Centrifugation Studies

The nanoemulsion system underwent centrifugation at a speed of 5000 rpm for 10 min and the system was examined for creaming or phase separation by visually observation of appearance [41].

##### Dilution Test

The formulation was diluted 50 and 100 times with distilled water, and the resulting mixtures were visually examined for clarity and any signs of phase separation [41].

##### Heating/Cooling Cycles

The impact of temperature fluctuations on the stability of nanoemulsion was seen through heating/cooling cycles. The temperature was changed 6 times between 8 and 40 °C, with storage lasting 48 h at each temperature [41].

#### 2.5.3. Determination of Drug Content

ACF from selected formulae was extracted in methanol using the sonication technique. After suitable dilution, the methanolic extract was analyzed for ACF content spectrophotometrically at a λ_max_ 276 nm [42].

#### 2.5.4. In Vitro Release Studies

The in vitro release study was conducted using the diffusion cell technique. The cellulose membrane was pre-soaked for 24 h in PBS to open the pores. The membrane was fitted between the donor and acceptor compartment and 2 g of the prepared formulae was placed on top of the membranes. The acceptor compartment was filled with 50 mL phosphate buffer pH 7.4 and continuously stirred using a magnetic stirrer at 100 rpm at 37 °C. Subsequently, 3 mL of dissolution media was withdrawn and replaced with fresh medium at the time intervals of 1, 2, 3, 4, 5, and 6 h. The amount of drug released was measured spectrophotometrically at λ_max_ 276 nm [43].

#### 2.5.5. Mathematical Modelling for In Vitro Release Studies

Zero-, first-, and second-order kinetics, as well as the Higuchi diffusion model and Hixson–Crowell cube root law, were used to choose the most suitable kinetic order for ACF release [44].

#### 2.5.6. Physical Investigation of Selected NE Formula

##### Differential Scanning Calorimetry (DSC)

DSC studies were performed for the drug, citronellol, Tween 80, Transcutol HP, and the selected formula with a DSC50 (Shimadzu, Kyoto, Japan; connected with thermal analyzer TA-501). Samples were heated in a hermetically sealed aluminium pan in a temperature range of −150 to 300 °C at a heating rate of 10 °C/min, under nitrogen flow of 20 mL/min [45].

##### Fourier-Transform Infrared Spectroscopy (FTIR)

FTIR spectra for the drug, citronellol, Tween 80, Transcutol HP, and the selected formula were determined using the KBr disc technique with an FTIR spectrophotometer (Bruker 22, Massachusetts, Billerica, UK) [45].

##### Transmission Electron Microscopy (TEM)

The morphological study of the prepared F1 was carried out by transmission electron microscopy. The sample was visualized by drying it on a carbon-coated grid and negatively staining it with an aqueous solution of phosphotungstic acid. After drying of the phosphotungstic acid, the sample was observed under TEM, (Joel JEM 1230, Tokyo, Japan) [46].

### 2.6. Antiproliferative and Pro-Apoptotic Activity Study against Melanoma Cell Line 

#### 2.6.1. Cell Culture

Human melanoma cells (A375) (ATCC^®^CRL-1619) were incubated with Dulbecco’s Modified Eagle’s Medium (Fisher Scientific, Oxford, UK), enriched with streptomycin, penicillin, and heat-inactivated FBS in a humidified and CO_2_ atmosphere at room temperature.

#### 2.6.2. Cytotoxicity Assay

Cell viability was assessed by SRB assay as reported [47]. Human melanoma cells were incubated with ACF, blank, and ACF NE at various concentrations for 72 h. Melanoma cells were mixed with TCA (10%) and maintained at 4 °C for 1 h, then washed with D.W. Sulforhodamine B (70 μL) (0.4% *w*/*v*) was added and kept for 10 min. Plates were washed by 1% acetic acid 3 times and left to dry overnight. TRIS (150 μL) was added to dissolve the protein-bound SRB stain. Lastly, absorbance was determined by a star Omega microplate reader (BMGLABTECH^®^-FLUO, Ortenberg, Germany) at 540 nm [48,49]. Cytotoxicity was expressed as (IC_50_), which was determined by plotting %viability against the concentration of the drug (five concentrations were used). IC_50_ values were calculated from the dose–response curve by the PRISM program.

#### 2.6.3. Flow Cytometry Study

##### Analysis of Cell Cycle Distribution

Cell cycle analysis was performed by flow cytometric assay as reported [49,50,51,52,53,54]. Melanoma cell line (A375) was treated with ACF NE and cisplatin at a dose of 5 µg/mL (positive control) for 48 h. After cell treatment with drugs for the specified period, trypsin was added to detach cells, cells washed with PBS (pH 7.4), then resuspended in 60% ethanol and incubated for 1 h. After fixation, cells were washed twice by PBS (pH 7.4) and incubated with PBS containing 50 µg/mL RNAase A and 10 µg/mL propidium iodide (PI) in the dark for 20 min. An FL2 signal detector was used to measure DNA contents at (λ_ex/em_ 535/617 nm) (ACEA Novo-cyte flow-cytometer, ACEA Biosciences, San Diego, CA, USA). Cell analysis was performed using ACEA Novo-Express software (ACEA Bio-sciences, San Diego, CA, USA).

##### Apoptosis Assay

Cell apoptosis was determined by flowcytometry using an Annexin V FITC apoptosis detection kit (Abcam Inc., Cambridge, UK). Melanoma cells (A375) were treated with ACF NE for 48 h while cisplatin (5 µg/mL) was used as a positive control. Briefly, cells were detached by trypsin then washed twice with PBS (pH 7.4), incubated in the dark with Annexin V-FITC/PI for 30 min at room temperature, and then injected using an ACEA Novocyte flowcytometer (ACEA Biosciences Inc., San Diego, CA, USA). FITC (λ_ex/em_ 488/530 nm) and PI (λ_ex/em_ 535/617 nm) fluorescent signals were detected with an FL1 and FL2 signal detector, respectively. Positive FITC and/or PI cells were measured and calculated using ACEA NovoExpress™ software Version 1.6.2 (ACEA Biosciences Inc., San Diego, CA, USA) [51,52,53,54].

### 2.7. In Silico Studies

Molecular docking studies were executed according to the reported procedures [55] on different proteins known to modulate cell proliferation and programmed cell death.

Firstly, molecular target prediction was carried out using the Swiss Target prediction webtool (http://www.swisstargetprediction.ch/, accessed on 4 July 2023) [56]. The most probable protein for ACF to interact with and to modulate the cell proliferation pathway was p38 mitogen-activated protein kinase (MAPK). The 3Dcrystal structures of proteins were obtained from the website (https://www.rcsb.org/, accessed on 4 July 2023) protein data bank. For MAPK, the code was 6m95 [57]; for TNF-α, 2az5 [58]; for BcL2, 4ieh [59]; and for COX-2, 3ln1 [60]. The downloaded 3D structures were prepared, and energy was minimized and readied for docking using Discover Studio 4.1 software. The entitled compounds ACF and citronellol were drawn using Chemdraw and then prepared for docking. The resulting docking scores were examined, and the best poses were selected.

### 2.8. Statistics

All of the experiments were replicated three times, and the data were presented as the mean ± standard deviation. GraphPad Prism 6 software (San Diego, CA, USA) was utilized to determine significant difference using one-way ANOVA (one-way analysis of variance) test (* *p* < 0.05, ** *p* < 0.01, *** *p* < 0.001 and **** *p* < 0.0001).

## 3. Results

### 3.1. Solubility Study

**Figure 1:**
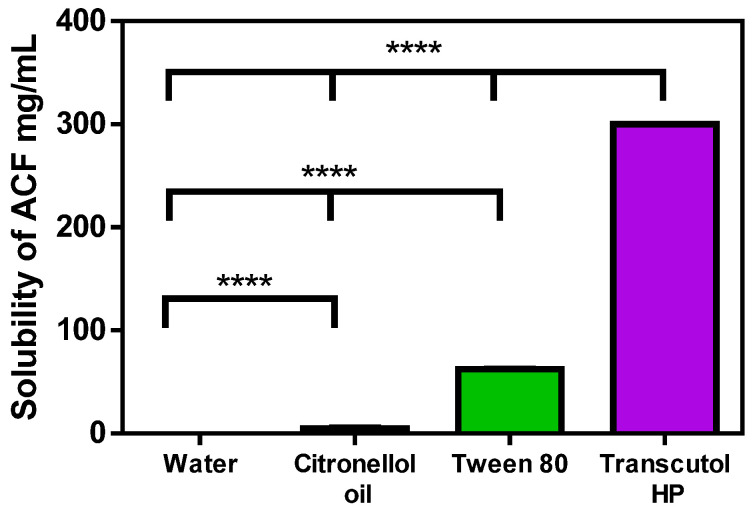
Solubility of ACF in different media. **** *p* < 0.0001.

The solubility of ACF in water, citronellol oil, Tween 80, and Transcutol HP were 0.071, 5.26, 62.6, and 300.2 mg/mL, respectively, as shown in Figure 1. It was observed that the solubility of ACF in citronellol oil was higher than in water. It was essential that the drugs had a higher oil solubility since this would help the nanoemulsion in keeping the drug in its solubilized state. As a result, fewer surfactants and co-surfactants are required for emulsification [61].

### 3.2. Estimation of Pseudoternary Phase Diagram

**Figure 2:**
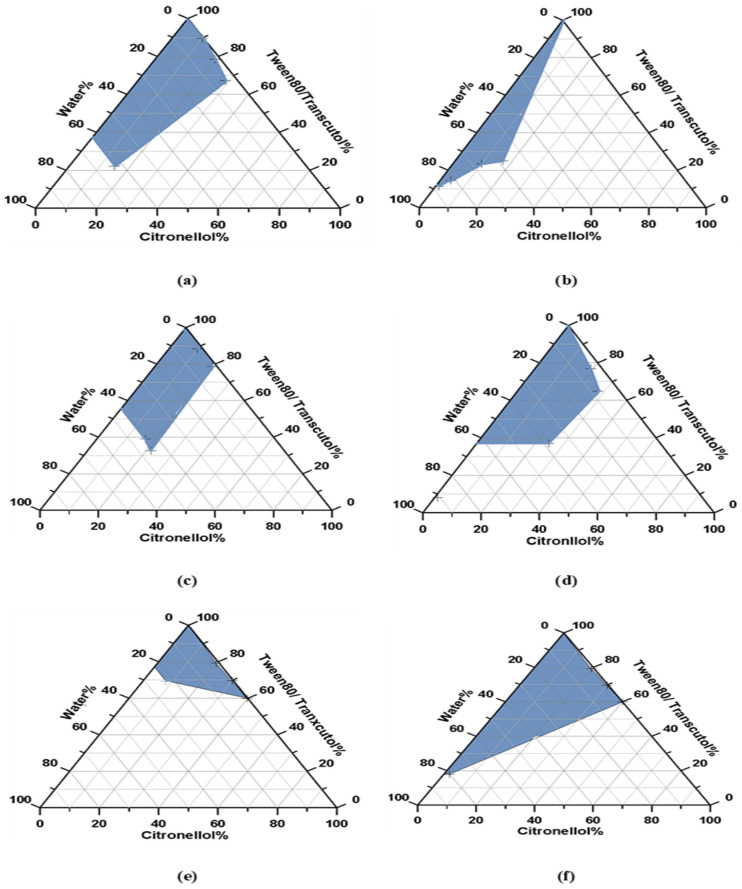
Pseudoternary phase diagrams of systems showing the highest emulsification region composed of citronellol (oil), Tween 80,Transcutol HP (S_mix_), and water with S_mix_. Ratios: (**a**) S_mix_ 1:1; (**b**) S_mix_ 2:1, (**c**) S_mix_ 3:1, (**d**) S_mix_ 4:1, (**e**) S_mix_ 7:1; and (**f**) S_mix_ 8:1.

Pseudoternary phase diagrams were established to identify emulsifying regions and to optimize the surfactant-to-co-surfactant ratio and the concentration of oil. The SAA concentration should be sufficient to stabilize droplets, achieve low interfacial tension, and prevent oil droplets from aggregating to produce NE [62]. Figure 2 showed that the SAA/co-SAA system was successful in lowering the interfacial tension between citronellol oil and water, making it easier to form NE droplets.

### 3.3. Preparation of Aceclofenac/Citronellol Oil NEs

Table 1 shows eight NE formulae prepared using Tween 80, Transcutol and citronellol oil in different ratios depending on the highest emulsification region in the pseudoternary phase diagram.

### 3.4. Characterization of Aceclofenac/Citronellol Oil NEs

#### 3.4.1. Droplet Size and Surface Charge Analysis

**Figure 3:**
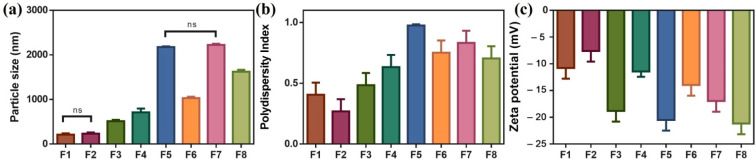
Particle size (**a**), polydispersity index (**b**), and zeta potential (**c**) of different ACF NE formulae.

Measuring the droplet size helps assess formulation homogeneity, stability, and bioavailability [63]. It was observed that F1, F2, and F3 had smaller particle sizes than other formulations that contained a higher percentage of citronellol oil. A decrease in polydispersity index (PDI) and an increase in homogeneity were observed with an increase in surfactant content. PDI ranged between 0.2693 and 0.4843 and all preparations showed negative charge zeta potential. The results are shown in Figure 3.

#### 3.4.2. Thermodynamic Stability Tests

The results of thermodynamic stability are presented in Table 2. The nanosized formulae F1, F2, and F3 passed all stability tests. They showed no signs of aggregation, creaming, or phase separation. Furthermore, these formulae exhibited stability under centrifuge stress and after heating–cooling cycle tests, with no precipitation or alteration in their physical appearance when they were diluted. F1, F2 and F3 were chosen for further evaluation studies.

#### 3.4.3. Determination of Drug Content

The drug content of the selected prepared formulae F1, F2, and F3 were 96.90% ± 0.11, 97.23% ± 0.27, and 84.43% ± 0.16, respectively. These data suggested proper oil and surfactant/cosurfactant selection in ACF NE preparation.

#### 3.4.4. In Vitro Release Studies 

**Figure 4:**
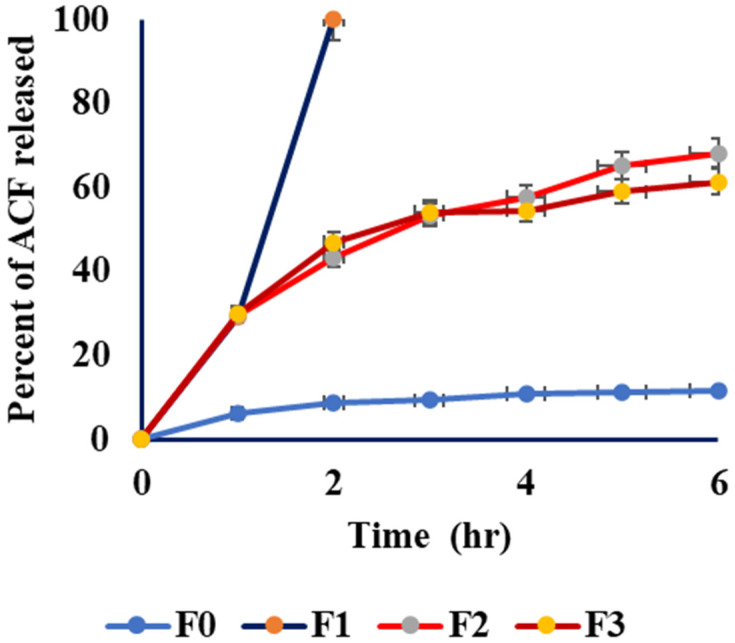
In vitro release study of ACF from F0, F1, F2, and F3.

F1 showed the maximum percentage of drug released after 2 h. This may be due to the lower particle size compared to other tested formulas (Figure 4).

#### 3.4.5. Mathematical Modelling for Release Studies

The in vitro release of ACF from the prepared nanoemulsions was characterized by determining the kinetic parameters. The release kinetics of the formula were evaluated based on the R^2^ values obtained. The results shown in Table 3 indicated that the drug release from F1 followed zero-order kinetics, as evidenced by the highest linearity observed in the plot. On the other hand, F2 and F3 followed the Higuchi diffusion model, representing that the amount of drug released was proportional to the square root of the total drug amount and the drug’s solubility [64].

#### 3.4.6. Physical Investigation of Selected NE Formula

##### Differential Scanning Calorimetry (DSC)

**Figure 5:**
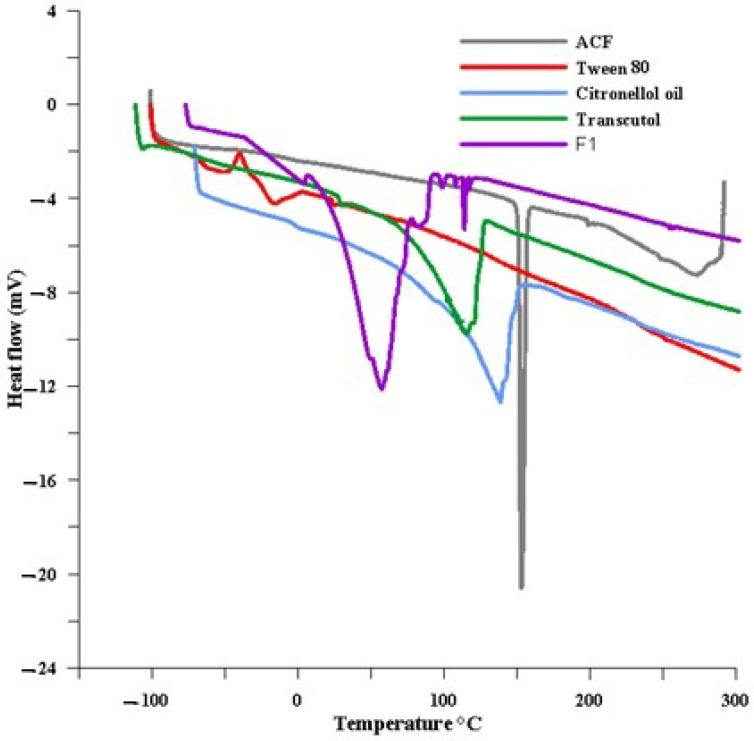
DSC thermogram of ACF, citronellol, the selected ACF NE formula (F1), Tween 80, and Transcutol HP.

Thermal properties of ACF, citronellol, the selected ACF NE formula (F1), Tween 80, and Transcutol HP were studied using DSC. The thermogram of ACF presents a sharp endotherm peak at 154.49 °C, indicating the crystalline nature of the drug. However, no distinctive ACF peak was identified in ACF NE (F1) thermogram at the studied temperature (Figure 5), confirming drug amorphization in nanoemulsion structure [65].

##### Fourier-Transform Infrared Spectroscopy (FTIR)

**Figure 6:**
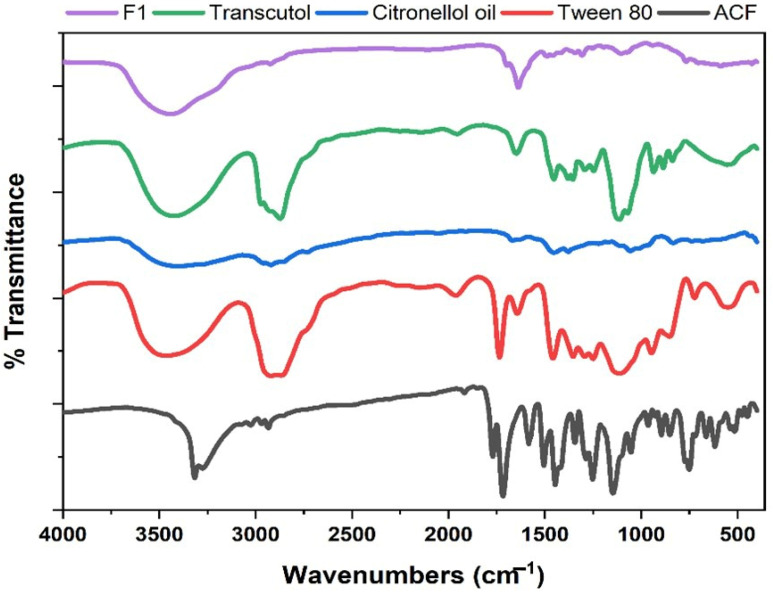
FTIR spectrum of the ACF, citronellol oil, the selected ACF NE formula (F1), Tween 80, and Transcutol HP.

FTIR spectra of the ACF, citronellol, the selected formula (F1), Tween 80, and Transcutol HP are depicted in Figure 6. FTIR spectra of pure ACF revealed the characteristic sharp peak at 3318 cm^−1^ indicating the 2° amine group, while its broadness indicates the presence of the acidic (OH) group. Also, there are two peaks at 1714 and 1770 cm^−1^, demonstrating the two-carbonyl group of the terminal chain. The aliphatic protons appeared just before 3000 cm^−1^ and the aromatic protons just after 3000 cm^−1^, and multiple phenyl ring bands appeared in the fingerprint region [66]. For citronellol, there is a broad peak between 3100 and 3500 cm^−1^ indicating the alcoholic OH group, while the small peak just before 3000 cm^−1^ indicates the aliphatic protons. The spectrum of ACF NE (F1) confirmed the same characteristic peaks of ACF with more broad bands indicating the homogenous solubility of the drug amorphously in the nanoemulsion structure [65].

##### Transmission Electron Microscopy (TEM)

**Figure 7:**
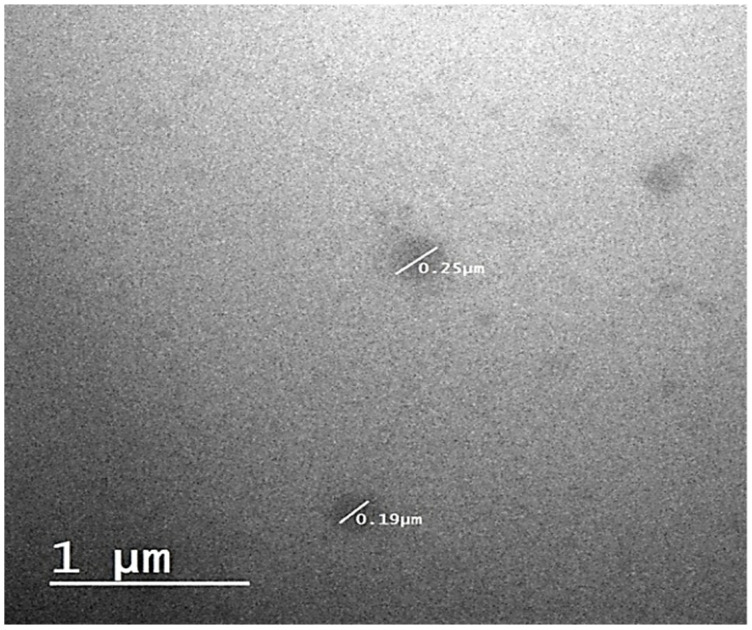
TEM image of the selected formula (F1).

Figure 7 shows the TEM image of (F1). It reveals spherical NE globules in the nanosize range, confirming the results obtained by the particle size analyzer. Spherical and non-aggregated globules in prepared formulae inferred the system stability.

### 3.5. Antiproliferative and Pro-Apoptotic Activity Study against Melanoma Cell Line

#### 3.5.1. Cytotoxicity Assay

**Figure 8:**
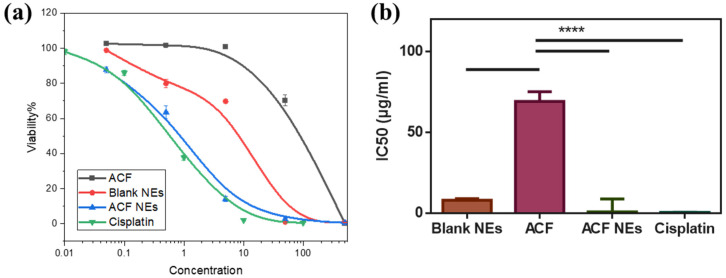
Cytotoxicity cell study determined by SRB assay. (**a**) Percentage of cell viability after treating melanoma cells with ACF, blank, ACF NE, and cisplatin. (**b**) IC50 values of ACF, ACF NE, and cisplatin. Values are the mean of three independent experiments (*n* = 3, **** *p* < 0.0001).

The cytotoxic effect of ACF, blank, and ACF NE was assessed against A375 human melanoma cell lines with SRB assay using cisplatin as a positive control. The cytotoxic efficacy was expressed as an IC_50_ value, representing the concentration of the drug needed to produce a 50% inhibition of cell growth after 48 h of incubation. The percentage of cell viability was significantly decreased with the increase in drug concentration. The blank manifested cytotoxic activity with an IC_50_ value of 8.04 µg/mL on human melanoma cell lines, which was attributed to the presence of citronellol oil [67]. Also, ACF exhibited cytotoxic activity with IC50 values of 69.27, but ACF NE’s IC_50_ was 0.81 µg/mL as compared to cisplatin (0.59 µg/mL) (Figure 8).

#### 3.5.2. Flow Cytometry Study

**Figure 9:**
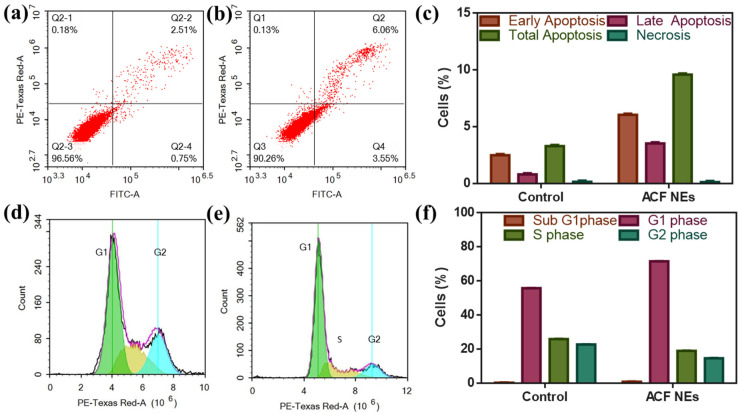
(**a**–**c**) Flow cytometric analysis for apoptosis in melanoma cells. Apoptosis was analyzed by annexin V staining and PI. Data shown are the percentage of cells counted. Human melanoma cells treated with (**a**) non-treated cells (control) and (**b**) ACF NE. (**c**) Percent of cell death by early apoptosis, late apoptosis and necrosis. Abbreviations: FITC-A: absorbance for fluorescein isothiocyanate. Q1: necrotic cells; Q2: late apoptotic cells; Q3: normal intact cells; and Q4: early apoptotic cells. (**d**–**f**) Flow cytometric analysis showing cell cycle distribution for (**d**) non-treated cells (control) and (**e**) ACF NE-treated cells. (**f**) Cell cycle distribution.

Flow cytometric analysis of ACF NE using annexin V and PI revealed 3.55% accumulation in the number of cells in the early (Q4) and 6.06% accumulation in the late stage (Q2) when compared to control, which presented 0.81% of cells accumulating in the early and 2.51% of cells in the late stage of apoptosis as shown in Figure 9a–c. In addition, cell cycle distribution was investigated by flow cytometry after treating cells with ACE NE at IC_50_ concentration. The results of cell cycle distribution presented in Figure 9d–f indicate the change in the cell cycle distribution after treatment, where there were 0.88% of the cells in the Sub-G1 phase in ACF NE but 0.28% in control due to apoptosis induction in cells treated by ACF NE, and a build-up of cells was seen in the G1 phase by (71.64%) compared to control (55.79%), with a remarkable decrease of cells in the S phase by (19%) and the G2 phase by (14.74%).

### 3.6. In Silico Studies

**Figure 10:**
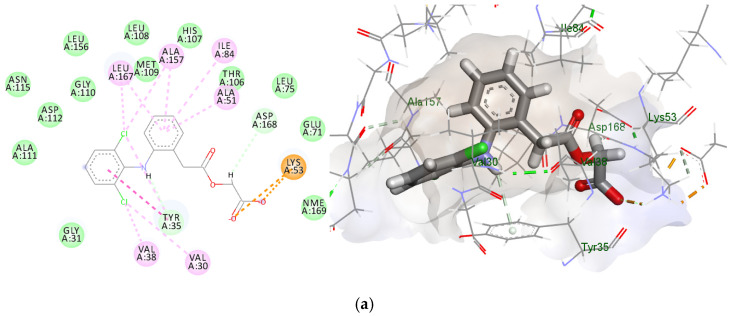

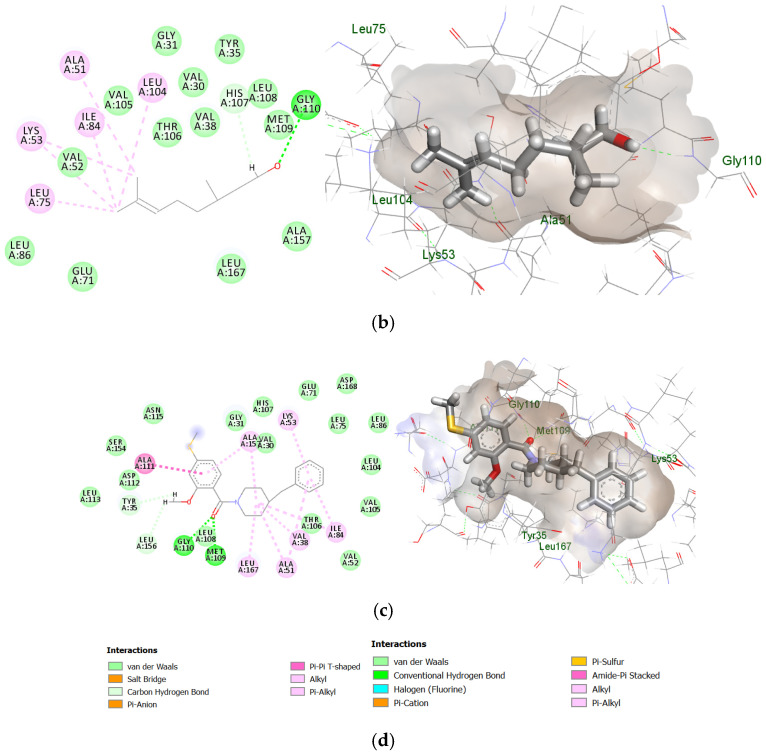
The 2D and 3D diagrams of aceclofenacvs.co-crystalized ligand J8s with their docking scores; (**a**) ACF = −7.08; (**b**) citronellol = −6.21; (**c**) J8s = −9.22 Kcal/mol; (**d**) types of interactions.

To suggest the molecular mechanism behind the cytotoxic and pro-apoptotic activity of ACF and citronellol incorporated in the NE, the web tool “SWISSTargetprediction” was used to predict the most probable macromolecular targets for ACF to be bioactive against [56]. MAPK was one of the highest probabilities, scoring 0.959. Thus, the docking started with MAPK or p38 mitogen-activated protein kinase. The 2D and 3D interaction diagrams and interaction scores are given in Figure 10.

BcL2 Interactions:

Both ACF and citronellol were docked against the active site of BcL2. The 2D and 3D interaction diagrams with scores are shown in Figure S1, giving docking scores of ACF = −5.08 and citronellol = −4.53 Kcal/mol.

TNF-α Interactions:

Docking results against TNF-α are depicted in Figure S2 with interaction scores for ACF = −5.97 and citronellol = −4.52, compared to the reference compound 307 with a score of −6.61 Kcal/mol.

COX-2 Interactions:

Docking of ACF and citronellol was performed against COX-2. Citronellol showed a comparable score to ACF but lower than the reference compound celecoxib. The interaction diagrams and docking scores are depicted in Figure S3 with docking scores of ACF = −8.16, citronellol = −5.89, and celecoxib = −9.95 Kcal/mol.

## 4. Discussion

This study demonstrates that the incorporation of citronellol oil enhances the solubility of the poorly water-soluble drug ACF, leading to improved formulation stability and enabling effective dose optimization. Tween^®^ 80 was selected for emulsification study as it showed good solubility potential, it has proved to be unaffected by the change modifications of pH and ionic strength, and it has low toxicity. By incorporating a suitable amount of cosurfactant (Transcutol HP), the emulsification process was enhanced, as it effectively reduced the interfacial tension, increased fluidity within the hydrocarbon region of the interfacial film, and minimized the bending stress at the interface [68].

To identify the regions of nanoemulsion formation and the optimal surfactant/cosurfactant ratios for the formation of a stable nanoemulsion system, phase diagrams were developed. In general, promising results were observed for nanoemulsification when using low oil concentrations. The incorporation of surfactant and cosurfactant further improved the emulsification process due to their increased hydrophilic characteristics. It was observed that using low oil content relative to the amount of surfactant or cosurfactant during NE formulation (F1, F2, and F3) decreased droplet size and enhanced the thermodynamic stability of NE. The results were in accordance with previous research by Nair et al. in their study on a sertraline self-nanoemulsion formulation. They interpreted the results in terms of oil, surfactant, and cosurfactant concentration effect on droplet size [69]. This observation was extracted from the characterization results of the nanosized thermodynamic stable formulae F1, F2, and F3. This result is consistent with two facts: the first is that the steric hindrance effect of the non-ionic surfactant Tween 80 builds up stable and clear NE with small particle size [70,71]. The second point is that there must be a minimum amount of oil in the NE formulation since a higher oil concentration results in larger droplets, which hinders the NE preparation and reduces its physical stability [72]. The use of non-ionic surfactants that have a consistent hydrophobic group composition may reduce the size of NE [61]. The utilization of Tween 80, a non-ionic surfactant, was associated with the negative zeta potential of all formulae. The polyoxyethylene group present in Tween 80 established hydrogen bonds with water molecules within the interfacial layer separating the oil and aqueous phases [73]. The high drug content percentage observed in the formulation can be attributed to the presence of low surface tension among the nanoemulsion (NE) droplets, which prevents their coalescence. This phenomenon was confirmed by the absence of phase separation, leading to enhanced drug retention and solubility within the formulation [72]. The chosen percentage of surfactant–co-surfactant blend (SAA) in the formulations was adequate for the formation of NEs with a clear and transparent appearance, while also ensuring improved stability. It is important to note that reducing particle size contributes to enhanced formulation stability by minimizing the occurrence of flocculation, gravitational force, and the impact of Brownian motion [24,74].

F1 NE achieved the maximum drug release after 2 h, which may be related to its smaller particle size compared to F2 and F3. This is in agreement with the smaller droplet size providing a larger surface area for drug dissolution. According to the Noyes–Whitney equation, the larger surface area increases the dissolution rate [75]. Furthermore, F1 exhibited the highest release percentage due to its low oil content and high S-mixture content. This composition results in a reduced requirement of free energy to create new interfaces between the lipid and water phases [76]. Moreover, DSC and FIIR confirmed the amorphous state of the drug in the NE and indicated the complete solubility of the drug in the NE base [77]. However, it conserved its thermodynamic stability that was achieved by the steric hindrance effect of Tween 80. Finally, the nanosized nonaggregate globules of formula F1 were confirmed by the TEM image. In medical applications, the size of nanoemulsion (NE) particles plays a crucial role in drug pharmacokinetics. The recommended range for the mean particle size of NEs in such applications is between 100 and 300 nm [78]. To prevent the obstruction of blood vessels, it is important to ensure that the size of nanoemulsion (NE) particles is not excessively large. Similarly, the particle size should not be excessively small to prevent indiscriminate and rapid absorption [79]. Compared to healthy tissue, the blood vessels in tumor tissue exhibit increased permeability. As the tumor rapidly grows, it either engulfs the existing blood vessels or stimulates the formation of new blood vessels in response to hypoxia. The tumor tissue is characterized by the development of newly formed blood vessels that exhibit permeability, enabling enhanced selective penetration of nanostructures and molecules larger than 40 KDa into the tumor stroma. Furthermore, the lack of proper lymphatic drainage in cancerous tissue plays a significant role in retaining nanoparticles [80,81].

In vitro cytotoxicity was performed by SRB Assay after treating melanoma cell line (A375) with ACF, ACF NE, cisplatin, and blank at various concentrations (0.05–500 µg/mL) for 48 h. Percentages of cell viability were measured, and then ic_50_ value (concentration required to produce 50% inhibition of cell viability) were calculated. The results revealed a decrease in the percentage of cell viability in a dose-dependent manner in the four treated groups. The melanoma cell line treated with cisplatin showed a higher decrease in cell viability compared to the other treated groups due to the potency of cisplatin as a chemotherapeutic drug. Despite cisplatin’s better cytotoxicity, it still has a lot of side effects that lead scientists look for better alternatives. However, NSAIDs such as ACF were proven to significantly decrease the risk of cancer development and metastasis, possibly through COX 2 inhibition [20]. Therefore, ACF can be used as a safer complementary therapy to chemotherapy. ACF NE (F1) presented a better cytotoxic effect on the melanoma cell line than ACF alone with (IC_50_ = 0.81 µg/mL) for ACF NE and (IC_50_ = 69.27 µg/mL) for ACF. ACF NE is more cytotoxic than ACF alone because of the synergistic effect of citronellol with ACF. It was reported previously in another study that citronellol exerted anti-cancer effects on different cell lines. Citronellol’s anti-proliferative effects were suggested to occur through different mechanisms such as changes in mitochondrial permeability, DNA fragmentation, increase in the production of ROS (reactive oxygen species), and caspase activation [31]. Therefore, our results revealed a significant decrease in the IC_50_ value of ACF from 69.27 µg/mL to 0.81 µg/mL (*p* < 0.0001) when formulated as a nanoemulsion, which is attributed to the synergistic effect of citronellol oil.

To prove the antiproliferative effect of ACF NE, Annexin-V-FITC Apoptosis Assay was performed. The current research highlighted that treating melanoma cells with ACF NE (F1) induced an increase in the percentage of cells that showed positive Annexin-V both in the early and late stages of apoptosis by nearly three-fold when compared to control. This finding suggests that the attributed cytotoxic activity of ACF NE (F1) is due to apoptosis induction. This is consistent with previous results that celecoxib, another cox 2 inhibitor, induces apoptosis through an intrinsic pathway by overexpression of the cleaved caspase 9 in the melanoma cell line [82].

Moreover, cell cycle distribution was performed after treating melanoma cells with ACF NE (F1) at its IC_50_ concentration. The results showed a significant build-up of cells in the G0-G1 phases almost three times higher than control due to apoptosis induction; in addition, the number of cells increased in the G1 phase compared to control, with a remarkable decrease of cells in the S phase and G2 phase due to cell arrest at G1 phase. These results emphasized the apoptotic effect of ACE (F1) by inducing G1 cell cycle arrest and apoptosis induction. This is consistent with another study that emphasized that citronellol significantly reduced cell proliferation by inducing cell cycle arrest at the G0-G1 phases in MDA-MB-231 cells [34]. Consequently, the antiproliferative effect of ACE NE (F1) was emphasized by the induction of cell cycle arrest at the G1 phase on A375 melanoma cells.

In silico docking was performed to postulate the mechanism behind the antiproliferative effect of ACF NE. Several mechanisms were suggested, such as MARK, BcL2, TNF-α, and COX-2 inhibition. MAPK is a well-known effector in cell proliferation pathways. Its altered expression is responsible for oncogenesis, progression, and emergence of drug resistance. Thus, its inhibition is considered a promising target in cancer treatment [77]. The docking results depicted in Figure 10 were comparable to the reference co-crystalized drug (J8s) with the 3D structure of MAPK. Both ACF and citronellol displayed promising binding interactions to the key amino acids in the active pocket, which suggests that one of the anticancer and apoptosis induction mechanisms may be MAPK inhibition. The key interactions were the amide–pi interaction of the phenyl ring of J8s with Ala111, two hydrogen bonds with Met109 and Gly110, the hydroxyl group interacting with Tyr35, the pyridazine ring being accommodated by the pocket formed by Leu167, Ala51, Val 38, and Thr106, and finally the pi–alkyl interactions of the second benzene ring with Ile34 and Lus53. Similarly, the chloro group of ACF showed pi interactions with Val30 and Val38, the amino group interacted via hydrogen bond with Tyr35, and the phenyl ring was accommodated in the pocket formed by Ala51, Leu167, and Ala157. The terminal carboxyl group interacted with Lys53.

For citronellol, the hydroxyl group formed a hydrogen bond with the key amino acid Gly110, while the remaining chain formed hydrophobic interactions with Ala51, Lys 53, Leu75, and Leu104.

BcL2 is a protein well known to control programmed cell deaths as its activation promotes cell survival and inhibits apoptosis [83,84]. The docking results against the BcL2 active site showed promising interactions with both ACF and citronellol compared to the reference drug, indicating their potential inhibition of this protein.

TNF-α is well known for its modulation of many important signal transduction pathways such as inflammation and cell proliferation, which are very important in controlling cancer cell survival [85]. Additionally, citronellol was reported to modulate TNF-α activity and inhibit cytokine production in mast cells [58,86], which led us to carry out docking studies of ACF and citronellol against it as a suggested mechanism for the pro-apoptotic activity of both drugs in the nanoemulsion.

Moreover, COX-2 enzyme (the major target for ACF for its anti-inflammatory properties) is known to be a promoter of immune suppression in melanoma, stimulate angiogenesis, help cancer cells evade apoptosis, and increase cell proliferation and cell invasiveness [87].

These abovementioned results obtained from docking studies on different proteins suggest that both drugs can have antiproliferative effects and pro-apoptotic mechanisms via inhibiting the above-mentioned targets, which proved beneficial in this nanoemulsion aiming to repurpose ACF in treating melanoma.

## 5. Conclusions

In this study, aceclofenac (ACF)/citronellol oil nanoemulsions (NEs) were successfully prepared using the sonication method. The formulations were subjected to extensive characterization to identify the optimal formula. The selected formula was then evaluated for its anticancer activity using a melanoma cell line, and in silico studies were conducted to gain insights into the suggested underlying mechanisms. The in vitro results highlighted the potential synergistic cytotoxic effect of ACF and citronellol oil NE, as evidenced by the induction of cell cycle arrest in A375 cells at a remarkably low dose. The in silico data suggested that the inhibition of MAPK, COX-2, BcL2, and TNF-α may contribute to the anticancer and apoptosis-inducing effects. Overall, the formulated ACF NE exhibited high potency and demonstrated promising therapeutic efficacy for the treatment of melanoma. Further research involving animal models is required to validate the antiproliferative and pro-apoptotic activity of ACF NE formulations.

## Figures and Tables

**Table 1:** Composition of the prepared nanoemulsion formulae.

Formulation Code *	Drug (gm)	Citronellol Oil (gm)	Tween 80 (gm)	Transcutol HP (gm)
F0 **	0.3	-	-	-
F1	0.3	1	4	1
F2	0.3	1	6	3
F3	0.3	1	8	1
F4	0.3	2	2	1
F5	0.3	3	4	3
F6	0.3	3	5	2
F7	0.3	4	3	3
F8	0.3	4	4	2

* Complete volume to 100 mL. ** F0: Pure drug suspension.

**Table 2:** Thermodynamic stability of prepared formulae.

Code	Centrifugation	Dilution Test	After Heating–Cooling Cycle
F1	Clear	Clear	Clear
F2	Clear	Clear	Clear
F3	Clear	Clear	Clear
F4	Creaming	Turbid	Turbid
F5	Phase Separation	Turbid	Turbid
F6	Creaming	Turbid	Turbid
F7	Phase Separation	Turbid	Turbid
F8	Phase Separation	Turbid	Turbid

Values are the mean of triplicate determination (*n* = 3) ± SD.

**Table 3:** Kinetic parameters for the in vitro drug release.

Formula	Mechanism of Release	Intercept	Slope	r *	k **	t_1/2(h)_ ***
F1	Zero-order	−6.883	49.950	0.973	49.950	1.001
F2	Diffusion	1.385	28.359	0.997	28.359	3.109
F3	Diffusion	4.298	25.449	0.978	25.449	3.860

* r: correlation coefficient. ** k: specific rate constant. *** t_1/2_: half-life.

## Data Availability

Data are contained within the article.

## Supplementary Materials

The following supporting information can be downloaded at: https://www.mdpi.com/article/10.3390/biomedicines11092531/s1, Figure S1: 2D and 3D Diagrams of Aceclofenac and Citronellol with Their Docking Scores in Bcl2 Binding Site, Figure S2: 2D and 3D Diagrams of Aceclofenac, Citronellol, And Co-crystalized Ligand 307 with Their Docking Scores, Figure S3: 2D and 3D diagrams of compounds under investigation Vs celecoxib with their docking scores.
