# Supplementary material for: Aceclofenac/Citronellol Oil Nanoemulsion Repurposing Study: Formulation, In Vitro Characterization, and In Silico Evaluation of Their Antiproliferative and Pro-Apoptotic Activity against Melanoma Cell Line
Source: Biomedicines. 2023 Sep 14;11(9):2531. doi: 10.3390/biomedicines11092531 (PMC10525854; doi:10.3390/biomedicines11092531)
Supplement: Supplementary file 1 [file biomedicines-11-02531-s001.zip › biomedicines-2591512-supplementary.pdf]

Aceclofenac/Citronellol Oil Nanoemulsion Repurposing study: Formulation, In-Vitro Characterization, and In-Silico Evaluation of their Antiproliferative and Pro-apoptotic Activity against Melanoma Cell Line

Supplemntary Figures:

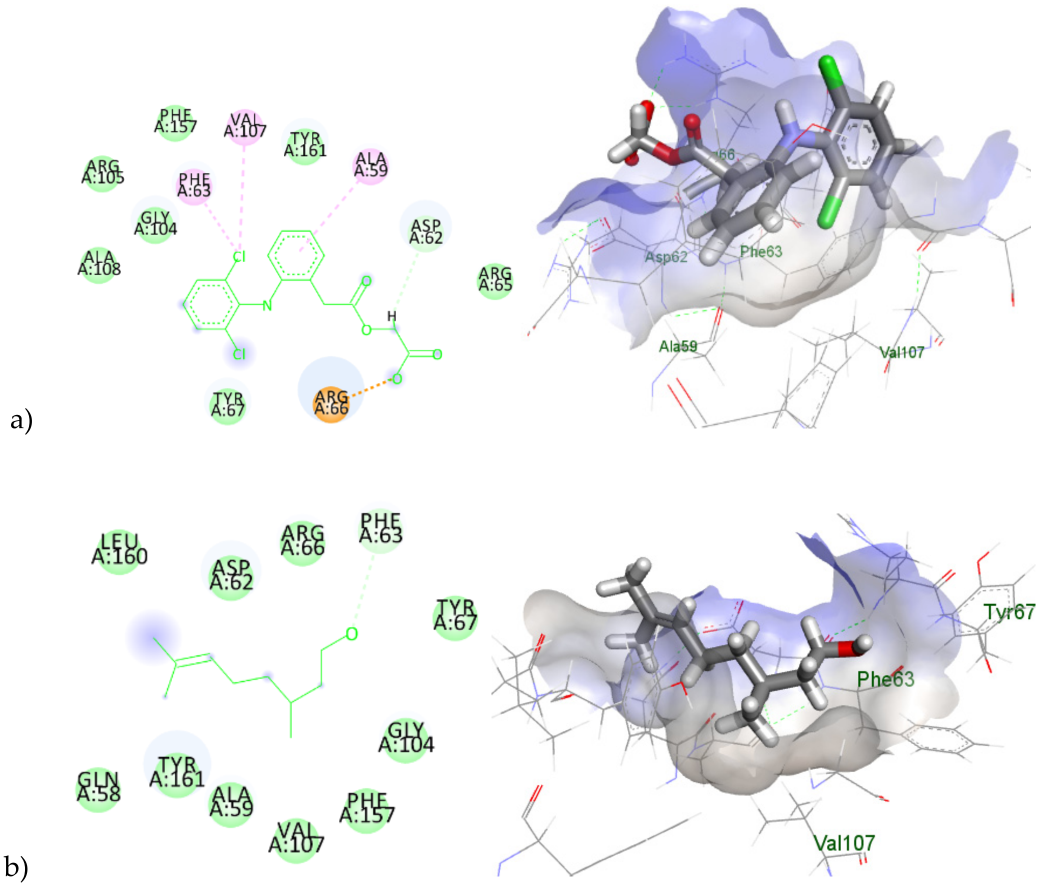

Figure S1. 2D and 3D Diagrams of Aceclofenac and Citronellol with Their Docking Scores in Bcl2 Binding Site; a) ACF= -5.08; b) Citronellol= -4.53 Kcal/mol.

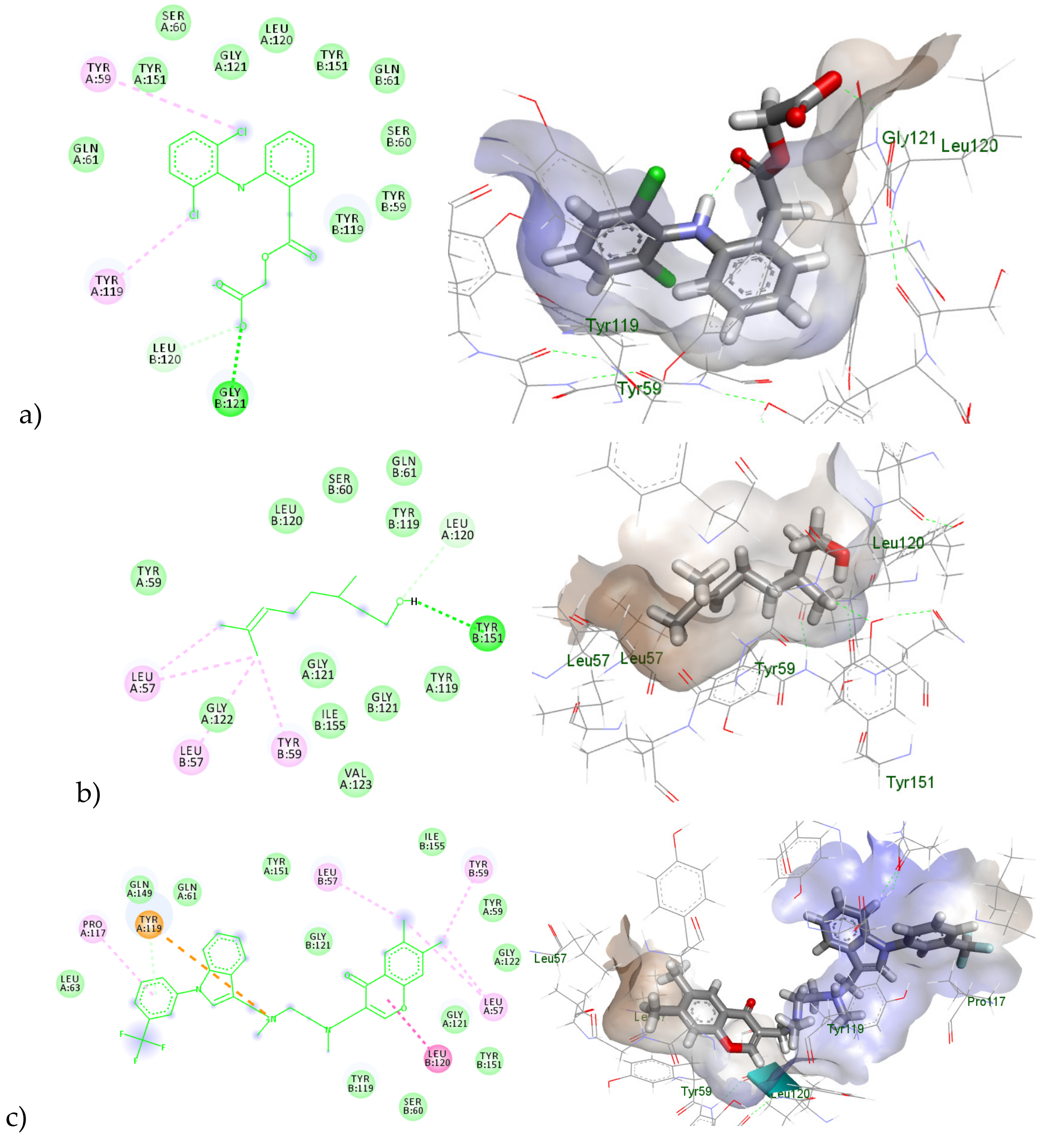

Figure S2. 2D and 3D Diagrams of Aceclofenac, Citronellol, And Co-crystalized Ligand 307 With Their Docking Scores; a) ACF= -5.97; b) Citronellol= -4.52; c) 307= -6.61 Kcal/mol.

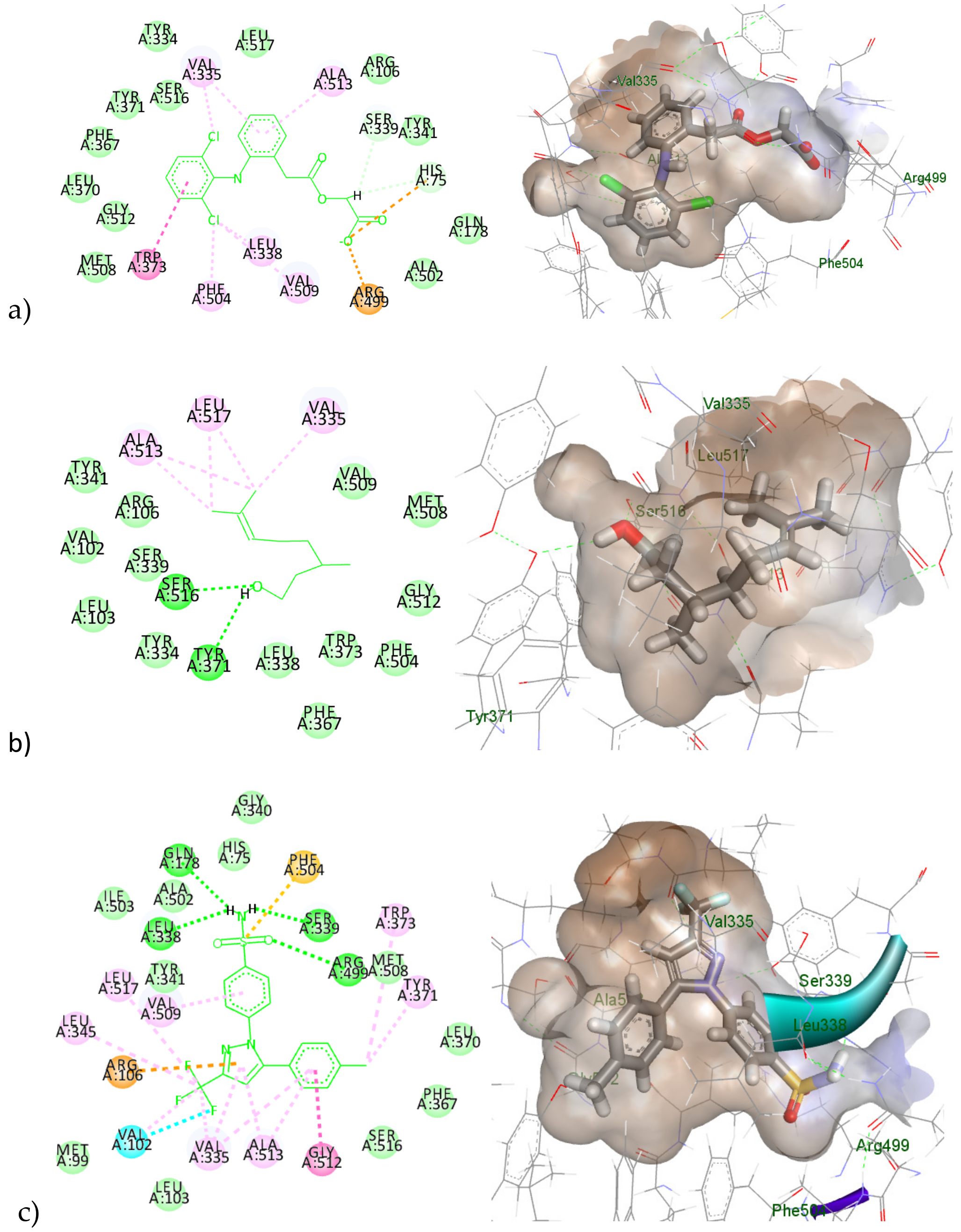

Figure S3. 2D and 3D diagrams of compounds under investigation Vs celecoxib with their docking scores; a) ACF= -8.16; b) Citronellol= -5.89; c) Celecoxib= -9.95 Kcal/mol.
